# Temporal trends in the prevalence, incidence, and mortality of cardiac amyloidosis in Korea over 12 years

**DOI:** 10.4178/epih.e2024078

**Published:** 2024-09-15

**Authors:** You-Jung Choi, Yun Jin Choi, Jieun Lee, Jah Yeon Choi, Geum Joon Cho, Jin Oh Na

**Affiliations:** 1 Division of Cardiology, Department of Internal Medicine, Korea University Guro Hospital, Seoul, Korea; 2 Biomedical Research Institute, Seoul National University Hospital, Seoul, Korea; 3 Biomedical Research Institute, Korea University Guro Hospital, Korea University College of Medicine, Seoul, Korea; 4 Department of Obstetrics and Gynecology, Korea University Guro Hospital, Seoul, Korea

**Keywords:** Amyloidosis, Asia, Cardiomyopathies, Epidemiology, Prognosis

## Abstract

**OBJECTIVES:**

This study investigated the prevalence, incidence, and prognosis of cardiac amyloidosis (CA) in Korea.

**METHODS:**

This retrospective nationwide population-based study used the Health Insurance Review and Assessment Service databases between 2008 and 2020. All patients diagnosed with amyloidosis were included, and those with a diagnosis of heart failure or cardiomyopathy were classified as having CA. Both the special code for amyloidosis (V121), which enables coverage of medical expenses, and the corresponding International Classification of Diseases, 10th revision codes for amyloidosis (E850- E854, E858, E859) were used to improve the reliability of amyloidosis diagnosis.

**RESULTS:**

Among 2,239 patients with amyloidosis, 758 met the criteria for CA (mean age, 64.4±11.9 years; 59.1% male). The mean age of patients with CA increased from 59.5±14.7 years in 2009 to 68.1±13.9 years in 2020. The incidence and prevalence increased from 0.09 (95% confidence interval [CI], 0.06 to 0.12) to 0.22 (95% CI, 0.18 to 0.27) per 100,000 person-years and 0.20 (95% CI, 0.16 to 0.25) to 1.30 (95% CI, 0.12 to 0.42) per 100,000 persons, respectively (all p<0.001). Patients with light-chain CA showed similar trends. In-hospital mortality decreased from 17.3% (95% CI, 9.23 to 29.6) to 6.10% (95% CI, 4.21 to 8.48) between 2009 and 2020. While age-specific in-hospital mortality was significantly higher in patients aged ≥70 years (p=0.004), no significant age-specific difference in in-hospital mortality was observed in patients with CA aged <70 years (p=0.981).

**CONCLUSIONS:**

The prevalence and incidence of CA have increased in Korea, predominantly affecting older individuals, particularly males. Notably, in-hospital mortality decreased significantly.

## GRAPHICAL ABSTRACT

[Fig f3-epih-46-e2024078]

## Key Message

This study investigated the epidemiology of cardiac amyloidosis in Korea from 2009 to 2020, revealing a significant increase in prevalence and incidence over time, along with a rise in the median age of patients. Notably, in-hospital mortality decreased substantially across all patients, reflecting improvements in treatment and management. However, among patients under 70 years of age, mortality due to cardiac amyloidosis showed no significant age-related differences, underscoring the importance of early detection and proactive treatment.

## INTRODUCTION

Cardiac amyloidosis (CA), which is characterized by the deposition of amyloid fibrils in the myocardium and leads to restrictive cardiomyopathy and heart failure, was previously regarded as a rare condition. However, there has been increasing recognition of this disease globally [1,2]. Advances in diagnostic modalities such as speckle-tracking echocardiography, bone tracer scintigraphy, and cardiac magnetic resonance imaging have improved the diagnostic rates of CA [3]. The emergence of new drugs such as tafamidis and daratumumab is also shifting the perception of CA from an intractable to a treatable disease, thereby promoting efforts to identify patients who could benefit from these novel therapies [4-8]. However, despite these advances, CA remains substantially underdiagnosed, which results in delayed diagnosis and poor outcomes [9,10]. If left untreated, the median survival of patients with CA is 1-2 years from the time of diagnosis [11]. Thus, early detection and treatment are critical for improved patient outcomes [10]. While the epidemiology and prognosis of CA have been studied in some countries, data on CA in Korea are limited. Thus, we aimed to fill this knowledge gap by investigating the nationwide, population-based epidemiological data of patients with CA in Korea.

## MATERIALS AND METHODS

### Study design and data sources

This retrospective, nationwide population-based study used data from the Health Insurance Review and Assessment Service (HIRA) database collected between 2008 and 2021. The HIRA database encompasses the insurance claims of approximately 98% of the Korean population, which equates to approximately 50 million individuals. The database contains the beneficiaries’ general information and healthcare service data, such as diagnoses, diagnostic tests, procedures, and prescriptions. All healthcare services were coded according to the 10th revision of the International Statistical Classification of Diseases and Related Health Problems (ICD-10).

### Study population and definitions

All patients diagnosed with amyloidosis between 2009 and 2020 were identified from the HIRA database. There is no consensus regarding the diagnosis of amyloidosis, particularly when utilizing claims data. However, in Korea, patients with amyloidosis are provided with a special code for the disease (V121) to allow for the coverage of medical expenses, since the disease is designated as rare and incurable. This approach enhances the accuracy of an amyloidosis diagnosis and ensures that patients receive appropriate medical care. Hence, to improve the reliability of an amyloidosis diagnosis in our analysis, we used both the special code for amyloidosis (V121) and the corresponding ICD-10 codes for amyloidosis (E850, E851, E852, E853, E854, E858, and E859). In addition, when amyloidosis coexisted with a plasma cell disorder (ICD-10 codes: C90 or D47.2), even in the absence of a V121 code for amyloidosis, patients diagnosed with plasma cell disorders were also considered to have amyloidosis where amyloidosis coexists, since the medical expenses for plasma cell disorder treatment can also be covered.

Light-chain (AL)-type amyloidosis is an acquired plasma cell disorder characterized by the pathological production of fibrillar proteins comprised of monoclonal light chains. Thus, among patients with amyloidosis, AL-type amyloidosis was defined as a plasma cell disorder that was subsequently treated with chemotherapy or hematopoietic stem cell transplantation after the initial diagnosis. The detailed diagnoses and classifications are listed in Supplementary Materials 1 and 2.

We considered patients with amyloidosis and a diagnosis of heart failure or cardiomyopathy (ICD-10 codes: I420, I4228, I425, I4288, I429, I431, I438, I5003, I5004, I5005, I5008, or I509) to have CA. We excluded patients with hypertrophic cardiomyopathy (I421, I4220, or I4221), alcoholic cardiomyopathy (I426), cardiomyopathy caused by drugs and other external agents (I427), or arrhythmogenic ventricular cardiomyopathy (I4280) (Supplementary Material 2). This definition of CA was based on previous studies [12].

### Clinical comorbidities and in-hospital mortality

Clinical comorbidities were defined using ICD-10 codes. The definitions of each comorbidity are listed in Supplementary Material 1 and have been previously validated [13,14]. To determine in-hospital mortality, we reviewed the discharge records. Patients whose records indicated they had died during hospitalization were classified as in-hospital deaths. If this event occurred within 1 year of their initial amyloidosis diagnosis, it was defined as 1-year in-hospital mortality.

### Statistical analysis

Differences in categorical variables (frequencies and percentages) were analyzed using the chi-square test or Fisher exact test. Continuous variables (mean± standard deviation) were analyzed using the Student t-test or Wilcoxon signed-rank sum test, depending on the distribution of the data. The annual prevalence and incidence were estimated based on cross-sectional data. To calculate the annual prevalence, the number of patients diagnosed with the disease was divided by the total population each year. The annual incidence was estimated by dividing the number of newly registered patients with amyloidosis by the entire population in the same year. To account for differences in age distribution over time, we employed direct age standardization to calculate annual age-standardized prevalence and incidence. The official dataset for the reference population was obtained from the Ministry of the Interior and Safety of Korea. For age standardization, we used the 2005 Korean standard population, accessible at https://jumin.mois.go.kr/ageStatMonth.do. To ensure a strict definition of incident cases of amyloidosis, we adopted a 1-year blanking period to exclude patients who had been diagnosed before they were registered in the HIRA database. The overall and annual prevalence and incidence rates were presented per 100,000 persons and person-years, respectively, with 95% confidence intervals (CIs).

For survival analysis, the Kaplan–Meier method was used to estimate the probabilities of event-free survival. Survival curves were plotted, and the log-rank test was used to evaluate the differences between groups based on sex and age. The Cox proportional hazards model was used to assess the crude hazard ratio and the impact of age groups on survival outcomes, after confirming the proportional hazards assumption based on Schoenfeld residuals. We considered the date of the initial diagnosis of amyloidosis as the index date. Each subject was followed up until either in-hospital death or, for censored individuals, the last recorded alive date based on the most recent hospital visit (outpatient or inpatient).

The results were expressed as hazard ratios with 95% CIs. We considered a two-sided p-value < 0.05 to indicate statistical significance. All statistical analyses were performed using SAS version 9.3 (SAS Institute Inc., Cary, NC, USA).

### Ethics statement

This study was approved by the Human Investigation Review Board of the Korean University Guro Hospital (2022GR0159), which waived the need for informed consent due to the retrospective study design and anonymity of the HIRA database.

## RESULTS

### Demographic characteristics

A total of 758 patients with CA were identified between 2009 and 2020. The demographic characteristics are presented in Table 1. The mean age of patients with CA was 64.4± 11.9 years, and 59.1% were male. The prevalence of hypertension, diabetes, and atrial fibrillation were 57.7%, 25.3%, and 12.3%, respectively.

The mean age of patients with CA increased annually from 59.5± 14.7 years in 2009 to 68.1± 13.9 years in 2020. The proportion of males increased from 54.7% in 2009 to 60.0% in 2020. As age increased, the prevalence of chronic diseases such as hypertension and diabetes also increased (Table 2). The baseline characteristics and annual trends of the patients with amyloidosis are listed in Supplementary Materials 3 and 4.

### Prevalence and incidence

**Figure 1.**
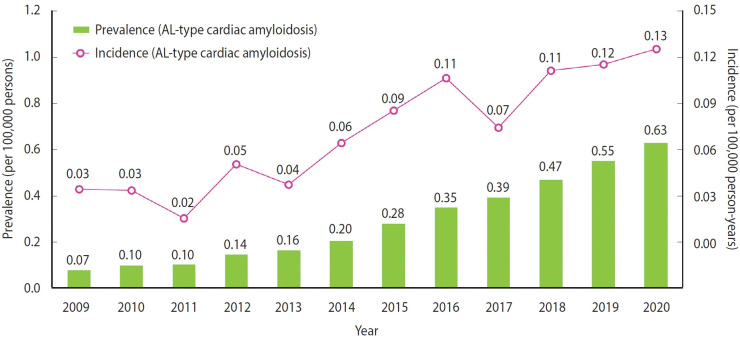
Trends in the prevalence and incidence of cardiac amyloidosis (CA) in the Korean population. The bar graph displays the prevalence of CA (per 100,000 persons) and the line graph indicates the incidence rate of CA (per 100,000 person-years) in the Korean population between 2009 and 2020. AL, light-chain.

Annual trends in the prevalence and incidence of CA in Korea over the 12-year study period are shown in Supplementary Material 5. The annual prevalence of CA rose from 0.20 (95% CI, 0.16 to 0.25) to 1.30 (95% CI, 1.20 to 1.42) per 100,000 persons in 2009 and 2020, respectively. A similar increase was seen in the incidence of newly diagnosed CA, from 0.09 (95% CI, 0.06 to 0.12) in 2009 to 0.22 (95% CI, 0.18 to 0.27) per 100,000 person-years in 2020 (all p-values < 0.001) (Figure 1). Comparable trends were observed for AL-type cardiac amyloidosis (Supplementary Material 6). The annual epidemiological trends of amyloidosis are shown in Supplementary Material 7.

### In-hospital mortality

**Figure 2.**
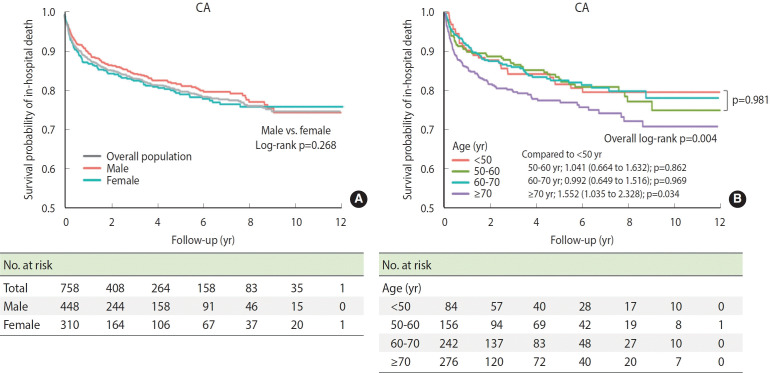
Kaplan–Meier curve for in-hospital mortality in Korean patients diagnosed with cardiac amyloidosis (CA). (A) The gray line represents the overall survival curve, while the pink and blue lines represent the survival curves for males and females. (B) Kaplan–Meier curves for in-hospital mortality by age groups; Values are presented as hazard ratio (95% confidence interval).

The in-hospital mortality of CA decreased by more than half, from 17.3% (95% CI, 9.23 to 29.6) in 2009 to 6.10% (95% CI, 4.21 to 8.48) in 2020 (p for trend < 0.001) (Supplementary Material 5). Among the incident cases, the 1-year in-hospital mortality also decreased from 36.4% in 2009 to 16.7% in 2020. Similar trends were observed in AL-type CA. In survival analysis, the Kaplan–Meier curves showed no significant difference in in-hospital mortality by sex (log-rank p= 0.268; Figure 2A). The in-hospital mortality of patients with amyloidosis was less than half that of those with CA (7.86%; 95% CI, 5.41 to 11.0 vs. 17.3%; 95% CI, 9.23 to 29.6 in 2009). The annual in-hospital mortality trend of amyloidosis is shown in Supplementary Material 7.

The age-specific in-hospital mortality among patients with CA was significantly higher in patients aged ≥ 70 years than in those < 50, 50-60, and 60-70 years (log-rank p= 0.004, Figure 2B). Interestingly, among patients with CA aged ≤ 70 years, no significant difference was observed in in-hospital mortality by age (logrank p= 0.981, Figure 2B), whereas there was a significant difference in in-hospital mortality by age in patients with amyloidosis (log-rank p< 0.001, Supplementary Material 8).

## DISCUSSION

In this study, we investigated the epidemiological data of patients with CA in Korea from 2009 to 2020. The main trends in temporal change were as follows: (1) the prevalence and incidence of CA in Korea exhibited an upward trend over time, (2) the median age of patients with CA increased steadily, and (3) in-hospital mortality, as well as the 1-year in-hospital mortality, decreased significantly over the study period. Notably, no significant differences were observed in in-hospital mortality by age among patients with CA who were < 70 years. Age-specific analyses showed that patients ≥ 70 years of age exhibited a considerably higher inhospital mortality than those aged < 70 years. To the best of our knowledge, this is the first epidemiological study of CA in an Asian population, and its results contribute to a better global understanding of CA.

The diagnosis of amyloidosis and the collection of accurate epidemiological data on the disease are challenging. However, there has been increasing recognition of CA in patients with common cardiac conditions such as atrial fibrillation, heart failure with preserved ejection fraction, and aortic stenosis [15-17]. In addition, new therapies are being developed for specific types of CA [4,6,7], and advancements in cardiac imaging, such as speckle-tracking echocardiography, magnetic resonance imaging, and nuclear cardiac scintigraphy, have facilitated the diagnosis of CA without biopsy [3]. Overall, our findings suggest an improvement in the inhospital mortality of patients with CA over the past decade, indicating that accuracy in the diagnosis and management of CA have improved over the years, leading to better patient outcomes.

Previous epidemiological studies have used both nationwide population cohort studies and single tertiary institution follow-ups to gather epidemiological data on amyloidosis. In Korea, Cho et al. [18] reported that the age-standardized prevalence of amyloidosis increased from 0.93 per 100,000 in 2006 to approximately 1.19 per 100,000 in 2015. In the current study, we observed a slightly higher prevalence of 3.19 per 100,000 in 2015. This difference may be attributable to the definitions used in our study. Unlike the previous report that focused only on the diagnosis of amyloidosis, we included patients diagnosed with plasma cell disorders and incorporated comprehensive diagnostic criteria. We propose that, by including these patients in our analysis, we were able to obtain a more accurate and comprehensive understanding of the prevalence of amyloidosis.

Our findings align with previous studies that showed a gradual improvement in survival for patients with AL-type CA [19,20]. However, while survival outcomes have shown a gradual improvement, early mortality within 6 months remains a significant challenge, with 28.1% of patients dying within 6 months [19]. In the current study, the in-hospital mortality of patients with AL-type CA decreased from 14.3% in 2009 to 7.4% in 2020, although most deaths occurred within the first years following diagnosis. The discrepancy in mortality rates may stem from the previous study’s focus on a high-risk cohort at a single tertiary care center, which may have involved different disease severities. In contrast, our study used nationwide data, encompassing a broader range of illness severity. In addition, our study reported in-hospital mortality rather than overall survival. Nevertheless, this high rate of in-hospital deaths, despite advancements in treatment and improved overall survival rates, suggests that late diagnosis and delayed treatment remain significant challenges. Notably, the in-hospital mortality rate among patients with cardiac involvement was nearly twice that of those with amyloidosis (approximately 3 to 8%) in this study, underscoring the critical impact of cardiac involvement.

Another study examined real-world treatment patterns, clinical outcomes, and costs for patients with AL-type amyloidosis. It found high rates of cardiac and renal failure and substantial costs, emphasizing the need for new therapeutic approaches to improve outcomes and reduce disease burden [21]. Despite increased diagnoses and reduced mortality, our study also emphasized the poor prognosis for individuals specifically affected by AL-type CA, reinforcing the challenging nature of cardiac involvement in AL-type amyloidosis.

Epidemiological studies on CA are limited by its rarity. Our findings provide valuable epidemiological data on patients diagnosed with CA in Korea, helping fill the knowledge gap. Interest in the epidemiology of CA has grown worldwide, with various studies reporting its incidence and prevalence. In 2019, Gilstrap et al. [12] reported the first contemporary estimate of the incidence and prevalence of CA among Medicare beneficiaries in the United States and concluded that, in hospitalized patients aged > 65 years, the incidence rate of CA was 17 per 100,000 person-years, with a prevalence of 55 per 100,000 persons. In Denmark, from 1998 to 2017, the incidence of CA increased, particularly in older males aged ≥ 65 years, with an incidence of 3.6 per 100,000 person-years and a decrease in mortality [22]. However, despite the significant burden of CA, data are scarce regarding its prevalence and incidence in the general population. Our study reported a prevalence and incidence rate of 1.30 per 100,000 persons and 0.22 per 100,000 person-years, respectively, in the general Korean population in 2020, lower than figures for elderly individuals aged 60-65 years or older. Furthermore, the global prevalence of amyloidosis, specifically the AL-type, across all countries was estimated from 3.2 per 100,000 in Brazil to 7.11 per 100,000 in Japan from 1999 to 2018 [23], which is significantly higher than our report. Differences in genetic background and the lack of pathological confirmation might have contributed to the under-enrollment of patients by V121 codes in Korea. Therefore, Korea must enhance efforts to identify undiagnosed patients and more rigorously confirm their diagnoses. Our study also highlighted a gradual increase in the prevalence and incidence rates of CA over time, as well as an increase in the average patient age. Although CA is rare, our findings underscore the need for continued monitoring of its prevalence and incidence in aging societies.

In addition to the epidemiological aspects of CA, interest in the clinical features and therapeutic options for CA has grown. A single-institutional study in Korea reported that, when CA was confirmed in patients with systemic amyloidosis, the mortality reached 58.1% over a 3-year follow-up period, which was significantly higher than the 37.3% mortality observed in patients without cardiac involvement [11]. One noteworthy finding from our study was that, although in-hospital mortality was higher for patients with CA aged ≥ 70 years, there was no age-related difference in in-hospital mortality among those below the age of 70 years. This implies that disease control may have a substantial impact on prognosis, especially in the presence of cardiac involvement, in patients with amyloidosis, although younger patients with amyloidosis have a better prognosis than older patients. Meanwhile, significant progress has been made in the diagnosis and management of AL-type amyloidosis over the past 40 years, resulting in improved survival and reduced early mortality [20]. While patients with transthyretin-CA currently face high medical costs and short survival due to diagnostic delays in real-world clinical practice [9,24], emerging therapeutic options for disease control offer favorable prospects for the future [25]. These observations provide a more optimistic outlook for patients with CA and highlight the importance of early diagnosis and treatment to improve prognosis and alleviate the economic burden, while new disease-modifying treatments are investigated and approved. To fully understand the impact of an aging society and advances in the management of amyloidosis, further research is warranted to elucidate the differences and changes in the characteristics of patients with amyloidosis over time. This research will provide a comprehensive understanding of how demographic shifts and therapeutic advances influence clinical outcomes in this population.

This study had some limitations. First, it relied on claims data from the HIRA database, which may lack accuracy and completeness in diagnostic coding. Therefore, the overall data trends should be emphasized rather than individual numbers. Second, it did not provide information on the type, severity, or genetic data of amyloidosis, which are crucial for prognosis and treatment decisions. Further studies should investigate the type of amyloidosis, treatment patterns, and clinical outcomes, including the use of novel therapies, to understand their impact on the prognosis and treatment response. Third, the study was limited to the Korean population, necessitating research on other populations for a global perspective. Finally, mortality data were confined to in-hospital deaths, potentially underrepresenting the overall mortality associated with CA. Consequently, while prevalence and incidence were age-standardized, in-hospital mortality was not. This limitation may affect the accuracy of our mortality comparisons between population groups due to unaccounted differences in age distribution. Furthermore, the absence of multivariate analysis in the Cox proportional hazards model limited our capacity to comprehensively interpret the influence of various factors on survival outcomes in this population. This limitation underscores the need for further research to investigate additional variables impacting survival.

In summanry, The prevalence of CA in Korea has increased significantly over the past decade, with an annual prevalence and incidence of 1.3 per 100,000 persons and 0.22 per 100,000 person-years, respectively, in 2020. These observations are consistent with the growing recognition of CA and highlight the need for increased awareness, early detection, and appropriate disease management, particularly in aging populations.

## Figures and Tables

**Figure f3-epih-46-e2024078:**
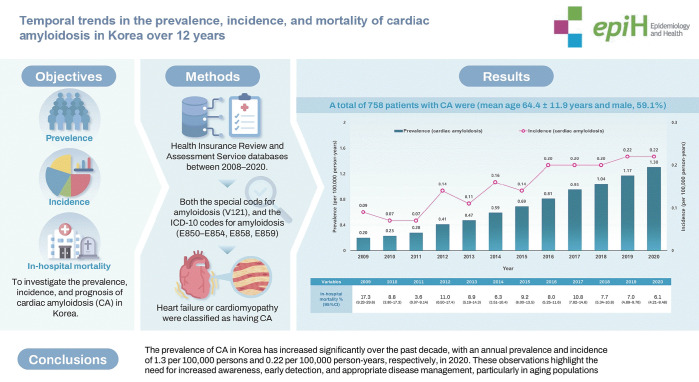


**Table 1.** Baseline characteristics of patients with cardiac amyloidosis in Korea (2009-2020)

Characteristics	Total (n=758)	Male (n=448)	Female (n=310)	p-value
Demographics				
Age, mean±SD (yr)	64.4±11.9	64.5±12.0	64.3±11.8	0.765
Age >65 yr	368 (48.6)	219 (48.9)	149 (48.1)	0.824
Male sex	448 (59.1)	448 (100)	-	
Low income^1^	29 (3.8)	16 (3.6)	13 (4.2)	0.323
Comorbidities				
Hypertension	437 (57.7)	260 (58.0)	177 (57.1)	0.797
Diabetes mellitus	192 (25.3)	120 (26.8)	72 (23.2)	0.267
Atrial fibrillation	93 (12.3)	63 (14.1)	30 (9.7)	0.070
Thromboembolism	67 (8.8)	46 (10.3)	21 (6.8)	0.096
Coronary artery disease	206 (27.2)	124 (27.7)	82 (26.5)	0.709
End-stage renal disease	41 (5.4)	29 (6.5)	12 (3.9)	0.119
Peripheral neuropathy	13 (1.7)	8 (1.8)	5 (1.6)	0.857
Carpal tunnel syndrome	39 (5.2)	21 (4.7)	18 (5.8)	0.493
Cancer	193 (25.5)	111 (24.8)	82 (26.5)	0.603

Values are presented as number (%). SD, standard deviation.

1 Low income was defined as participation in the medical aid program or a monthly income within the lowest 25% of the population.

**Table 2.** Annual trends of the baseline characteristics of patients in Korea with cardiac amyloidosis

Variables	2009	2010	2011	2012	2013	2014	2015	2016	2017	2018	2019	2020
Demographics												
Total (n)	75	91	112	164	191	239	283	339	398	441	499	560
Age, mean±SD (yr)	59.5±14.7	59.2±14.1	62.0±14.2	61.8±14.0	62.3±13.9	63.3±13.9	63.8±14.0	64.9±13.7	65.8±13.8	66.4±13.8	67.1±13.8	68.1±13.9
Age >65 yr	24 (32.0)	25 (27.5)	39 (34.8)	57 (34.8)	63 (33.0)	87 (36.4)	102 (36.0)	134 (39.5)	166 (41.7)	189 (42.9)	216 (43.3)	260 (46.4)
Male sex	41 (54.7)	53 (58.2)	66 (58.9)	96 (58.5)	114 (59.7)	139 (58.2)	166 (58.7)	199 (58.7)	236 (59.3)	256 (58.1)	291 (58.3)	336 (60.0)
Low income^1^	2 (2.7)	2 (2.2)	3 (2.7)	6 (3.7)	8 (4.2)	9 (3.8)	11 (3.9)	12 (3.5)	16 (4.0)	15 (3.4)	20 (4.0)	24 (4.3)
Comorbidities												
Hypertension	38 (50.7)	44 (48.4)	63 (56.3)	88 (53.7)	95 (49.7)	129 (54.0)	153 (54.1)	187 (55.2)	217 (54.5)	241 (54.7)	279 (55.9)	309 (55.2)
Diabetes mellitus	14 (18.7)	14 (15.4)	21 (18.8)	32 (19.5)	44 (23.0)	56 (23.4)	68 (24.0)	74 (21.8)	82 (20.6)	101 (22.9)	111 (22.2)	140 (25.0)
Atrial fibrillation	3 (4.0)	5 (5.5)	9 (8.0)	11 (6.7)	14 (7.3)	16 (6.7)	20 (7.1)	24 (7.1)	38 (9.6)	43 (9.8)	61 (12.2)	71 (12.7)
Thromboembolism	11 (14.7)	13 (14.3)	15 (13.4)	20 (12.2)	19 (10.0)	20 (8.4)	22 (7.8)	30 (8.9)	35 (8.8)	40 (9.1)	44 (8.8)	51 (9.1)
Coronary artery disease	9 (12.0)	10 (11.0)	12 (10.7)	15 (9.2)	15 (7.9)	14 (5.9)	17 (6.0)	23 (6.8)	28 (7.0)	32 (7.3)	36 (7.2)	43 (7.7)
Cardiomyopathy	2 (2.7)	3 (3.3)	4 (3.6)	6 (3.7)	5 (2.6)	6 (2.5)	6 (2.1)	7 (2.1)	7 (1.8)	8 (1.8)	8 (1.6)	8 (1.4)
End-stage renal disease	1 (1.3)	1(1.1)	1 (0.9)	1 (0.6)	1 (0.5)	2 (0.8)	2 (0.7)	3 (0.9)	3 (0.8)	3 (0.7)	4 (0.8)	4 (0.7)
Peripheral neuropathy	15 (20.0)	18 (19.8)	24 (21.4)	37 (22.6)	44 (23.0)	58 (24.3)	70 (24.7)	81 (23.9)	95 (23.9)	104 (23.6)	130 (26.1)	146 (26.1)
Carpal tunnel syndrome	1 (1.33)	1 (1.1)	1 (0.9)	2 (1.2)	3 (1.6)	4 (1.7)	4 (1.4)	5 (1.5)	4 (1.0)	4 (0.9)	3 (0.6)	3 (0.5)
Cancer	24 (14.9)	13 (11.6)	32 (24.8)	39 (24.1)	35 (21.7)	39 (18.2)	39 (20.3)	49 (20.3)	53 (22.8)	54 (23.0)	57 (29.1)	59 (28.9)

Values are presented as number (%). SD, standard deviation.

1 Low income was defined as participation in the medical aid program or having a monthly income within the lowest 25% of the population.

## Supplementary materials

Supplementary Material 1. List of International Statistical Classification of Diseases and Related Health Problems, 10th revision code for diagnosis.

Supplementary Material 2. Definition of amyloidosis and cardiac involvement.

Supplementary Material 3. Baseline characteristics of amyloidosis.

Supplementary Material 4. Baseline characteristics of amyloidosis.

Supplementary Material 5. Annual trend of prevalence, incidence, and in-hospital mortality of cardiac amyloidosis

Supplementary Material 6. Trends in the prevalence and incidence of AL-type cardiac amyloidosis in the Korean population.

Supplementary Material 7. Annual trend of prevalence, incidence, and in-hospital mortality of amyloidosis.

Supplementary Material 8. Kaplan–Meier curve for mortality in patients diagnosed with amyloidosis in Korea.
