# Supplementary material for: Temporal trends in the prevalence, incidence, and mortality of cardiac amyloidosis in Korea over 12 years
Source: Epidemiol Health. 2024 Sep 15;46:e2024078. doi: 10.4178/epih.e2024078 (PMC11832237; doi:10.4178/epih.e2024078)
Supplement: Supplementary Material 1. — List of International Statistical Classification of Diseases and Related Health Problems, 10th revision code for diagnosis. [file epih-46-e2024078-Supplementary-1.docx]

**Supplemental Material 1.** List of International Statistical Classification of Diseases and Related Health Problems, 10^th^ revision code for diagnosis.

| **ICD-10 codes** | **Diagnosis** |
| --- | --- |
| **Amyloidosis** |  |
| E85 | Amyloidosis |
| E85.0 | Non-neuropathic heredofamilial amyloidosis |
| E85.1 | Neuropathic heredofamilial amyloidosis |
| E85.2 | Heredofamilial amyloidosis, unspecified |
| E85.3 | Secondary systemic amyloidosis |
| E85.4 | Organ-limited amyloidosis |
| E85.8 | Other amyloidosis |
| E85.9 | Amyloidosis, unspecified |
| **Plasma cell disorder** |  |
| C90 | Multiple myeloma and malignant plasma cell neoplasms |
| C90.0 | Multiple myeloma |
| C90.1 | Plasma cell leukemia |
| C90.2 | Extramedullary plasmacytoma |
| C90.3 | Solitary plasmacytoma |
| D47.2 | Monoclonal gammopathy of undetermined significance (MGUS) |
| **Cardiomyopathy** |  |
| **Inclusion** |  |
| I42.0 | Dilated cardiomyopathy |
| I42.5 | Other restrictive cardiomyopathies |
| I42.8 | Other hypertrophic cardiomyopathy |
| I42.9 | Cardiomyopathy, unspecified |
| I43 | Cardiomyopathy in diseases classified elsewhere |
| I43.1 | Cardiomyopathy in metabolic diseases |
| I43.8 | Cardiomyopathy in other diseases classified elsewhere |
| I50 | Congestive heart failure |
| **Exclusion** |  |
| I42.1 | Obstructive hypertrophic cardiomyopathy |
| I42.20 | Nonobstructive hypertrophic cardiomyopathy |
| I42.21 | Apical hypertrophic cardiomyopathy |
| I42.6 | Alcoholic cardiomyopathy |
| I42.7 | Cardiomyopathy due to drugs and other external agents |
| I42.80 | Arrhythmogenic ventricular cardiomyopathy |
| **Comorbidities** |  |
| Hypertension | I10-I13, I15 |
| Diabetes mellitus | E11-E14 |
| Atrial fibrillation | I48 |
| Thromboembolism | I63, I64, G45.8, G45.9, I74 |
| Coronary artery disease | I20-I25 |
| End-stage renal disease | N18.5, Z49 |
| Peripheral neuropathy | E85.1, G60.0, G60.8, G60.9 |
| Carpal tunnel syndrome | G56.0 |
