# Supplementary material for: Temporal trends in the prevalence, incidence, and mortality of cardiac amyloidosis in Korea over 12 years
Source: Epidemiol Health. 2024 Sep 15;46:e2024078. doi: 10.4178/epih.e2024078 (PMC11832237; doi:10.4178/epih.e2024078)
Supplement: Supplementary Material 2. — Definition of amyloidosis and cardiac involvement. [file epih-46-e2024078-Supplementary-2.docx]

**Supplemental Material 2.** Definition of amyloidosis and cardiac involvement.

| **Diagnosis** | **Definition** |
| --- | --- |
| **Amyloidosis** | - Primary diagnosis of E850, E851, E852, E853, E854, E858, E859 and RID code of V121  - Primary and/or secondary diagnosis of E850, E851, E852, E853, E854, E858, E859 and primary and/or secondary diagnosis of C990, C901, C902, C903, D472 |
| **AL-type amyloidosis** | - Primary diagnosis of amyloidosis and/or RID code of V121 with the following criteria;  1) primary and/or secondary diagnosis of C990, C901, C902, C903, D472; or 2) chemotherapy^*^ or hematopoietic stem cell transplant of plasma cell disorder/amyloidosis  - Exclusion: Non-neuropathic heredofamilial amyloidosis (E85.0x), Neuropathic heredofamilial amyloidosis (E85.1x), Heredofamilial amyloidosis, unspecified (E85.2x), Secondary systemic amyloidosis (E85.3x) |
| **Cardiac amyloidosis** | Amyloidosis with I420, I4228, I425, I4288, I429, I431, I438, I5003, I5004, I5005, I5008, I509  - Exclusion: hypertrophic cardiomyopathy (I421, I422), Alcoholic cardiomyopathy (I426), Cardiomyopathy due to drugs and other external agents (I427), Arrhythmogenic ventricular cardiomyopathy (I4280) |

^*^Chemotherapy with bendamustine, bortezomib, carfilzomib, cyclophosphamide, dexamethasone, prednisone, doxycycline, lenalidomide, melphalan, pomalidomide, and thalidomide on or after the first amyloidosis diagnosis during the study period.
