# Supplementary material for: Temporal trends in the prevalence, incidence, and mortality of cardiac amyloidosis in Korea over 12 years
Source: Epidemiol Health. 2024 Sep 15;46:e2024078. doi: 10.4178/epih.e2024078 (PMC11832237; doi:10.4178/epih.e2024078)
Supplement: Supplementary Material 3. — Baseline characteristics of amyloidosis. [file epih-46-e2024078-Supplementary-3.docx]

**Supplemental Material 3. Baseline characteristics of amyloidosis.**

| **Variables** | **Amyloidosis** |
| --- | --- |
| **Demographics** |  |
| Number | 2,239 |
| Age, years | 61.5 ± 13.1 |
| Age >65 years, n (%) | 913 (40.8) |
| Sex, Male, n (%) | 1,224 (54.7) |
| Low income^*^ | 93 (4.2) |
| **Comorbidities, n (%)** |  |
| Hypertension | 1100 (49.1) |
| Diabetes mellitus | 510 (22.8) |
| Atrial fibrillation | 130 (5.8) |
| Thromboembolism | 171 (7.6) |
| Coronary artery disease | 367 (16.4) |
| End-stage renal disease | 126 (5.6) |
| Peripheral neuropathy | 26 (1.2) |
| Carpal tunnel syndrome | 81 (3.6) |
| Cancer | 493 (22.0) |

^*^Low income was defined as either being a participant in the medical aid program or having a monthly income within the lowest 25% of the population.
