# Supplementary material for: Temporal trends in the prevalence, incidence, and mortality of cardiac amyloidosis in Korea over 12 years
Source: Epidemiol Health. 2024 Sep 15;46:e2024078. doi: 10.4178/epih.e2024078 (PMC11832237; doi:10.4178/epih.e2024078)
Supplement: Supplementary Material 4. — Baseline characteristics of amyloidosis. [file epih-46-e2024078-Supplementary-4.docx]

**Supplemental Material 4. Baseline characteristics of amyloidosis.**

| **Variables** | **2009** | **2010** | | **2011** | | **2012** | | **2013** | | **2014** | | **2015** | | **2016** | | **2017** | | **2018** | | **2019** | | **2020** |
| --- | --- | --- | --- | --- | --- | --- | --- | --- | --- | --- | --- | --- | --- | --- | --- | --- | --- | --- | --- | --- | --- | --- |
| **Demographics** |  |  | |  | |  | |  | |  | |  | |  | |  | |  | |  | |  |
| Number | 420 | 499 | | 587 | | 725 | | 845 | | 999 | | 1,142 | | 1,320 | | 1,484 | | 1,639 | | 1,752 | | 1,879 |
| Prevalence (100,000 persons) | 1.28 | 1.49 | | 1.82 | | 2.09 | | 2.45 | | 2.77 | | 3.17 | | 3.53 | | 3.87 | | 4.10 | | 4.36 | | 4.36 |
| Age, years | 57.4±13.7 | 58.1±13.5 | | 59.2±13.5 | | 60.1±13.2 | | 60.3±13.3 | | 61.0±13.4 | | 61.9±13.3 | | 62.5±13.1 | | 63.2±13.2 | | 63.6±13.3 | | 64.2±13.3 | | 65.0±13.3 |
| Age >65 y, n (%) | 126 (30.0) | 147 (29.5) | | 177 (30.2) | | 224 (30.9) | | 252 (29.8) | | 312 (31.2) | | 365 (32.0) | | 429 (32.5) | | 500 (33.7) | | 555 (33.9) | | 602 (34.4) | | 674 (35.9) |
| Sex, Male, n (%) | 222 (52.9) | 261 (52.3) | | 315 (53.7) | | 389 (53.7) | | 457 (54.1) | | 531 (53.2) | | 601 (52.6) | | 706 (53.6) | | 798 (53.5) | | 867 (52.9) | | 927 (52.9) | | 1013 (53.9) |
| Low incomes^*^ | 19 (5.8) | 16 (4.5) | | 23 (3.2) | | 32 (4.4) | | 40 (4.7) | | 41 (4.1) | | 49 (4.3) | | 54 (4.1) | | 61 (4.1) | | 61 (3.7) | | 69 (3.9) | | 77 (4.1) |
| **Comorbidities, n (%)** |  | |  | |  | |  | |  | |  | |  | |  | |  | |  | |  | |
| Hypertension | 156 (37.1) | 190 (38.1) | | 243 (41.4) | | 313 (43.2) | | 352 (41.7) | | 422 (42.2) | | 487 (42.6) | | 576 (43.6) | | 647 (43.6) | | 712 (43.4) | | 763 (43.6) | | 824 (43.9) |
| Diabetes mellitus | 63 (15.0) | 70 (14.0) | | 104 (17.7) | | 141 (19.5) | | 165 (19.5) | | 204 (20.4) | | 239 (20.9) | | 261 (19.8) | | 287 (19.3) | | 324 (19.8) | | 343 (19.6) | | 391 (20.8) |
| Atrial fibrillation | 10 (2.4) | 12 (2.4) | | 21 (3.6) | | 25 (3.5) | | 28 (3.3) | | 30 (3.0) | | 35 (3.1) | | 41 (3.1) | | 58 (3.9) | | 66 (4.0) | | 87 (5.0) | | 96 (5.1) |
| Thromboembolism | 32 (7.6) | 39 (7.8) | | 41 (7.0) | | 50 (6.9) | | 55 (6.5) | | 66 (6.6) | | 74 (6.5) | | 97 (7.4) | | 107 (7.2) | | 120 (7.3) | | 130 (7.4) | | 140 (7.5) |
| Coronary artery disease | 47 (11.2) | 59 (11.8) | | 74 (12.6) | | 98 (13.5) | | 110 (13.0) | | 136 (13.6) | | 157 (13.8) | | 181 (13.7) | | 197 (13.3) | | 213 (13.0) | | 236 (13.5) | | 260 (13.8) |
| End-stage renal disease | 1 (0.2) | 3 (0.6) | | 13 (2.2) | | 19 (2.6) | | 33 (3.9) | | 49 (4.9) | | 64 (5.6) | | 70 (5.3) | | 75 (5.1) | | 76 (4.6) | | 77 (4.4) | | 77 (4.1) |
| Peripheral neuropathy | 2 (0.5) | 4 (0.8) | | 5 (0.9) | | 6 (0.8) | | 5 (0.6) | | 5 (0.5) | | 5 (0.4) | | 4 (0.3) | | 9 (0.6) | | 13 (0.8) | | 21 (1.2) | | 22 (1.2) |
| Carpal tunnel syndrome | 7 (1.7) | 7 (1.4) | | 9 (1.5) | | 12 (1.7) | | 19 (2.3) | | 26 (2.6) | | 31 (2.7) | | 33 (2.5) | | 38 (2.6) | | 45 (2.8) | | 52 (3.0) | | 57 (3.0) |
| Cancer | 48 (11.4) | 56 (11.2) | | 82 (14.0) | | 114 (15.7) | | 139 (16.5) | | 167 (16.7) | | 196 (17.2) | | 228 (17.3) | | 269 (18.1) | | 305 (18.6) | | 339 (19.4) | | 378 (20.1) |

^*^Low income was defined as either being a participant in the medical aid program or having a monthly income within the lowest 25% of the population.
