# Supplementary material for: Temporal trends in the prevalence, incidence, and mortality of cardiac amyloidosis in Korea over 12 years
Source: Epidemiol Health. 2024 Sep 15;46:e2024078. doi: 10.4178/epih.e2024078 (PMC11832237; doi:10.4178/epih.e2024078)
Supplement: Supplementary Material 5. — Annual trend of prevalence, incidence, and in-hospital mortality of cardiac amyloidosis [file epih-46-e2024078-Supplementary-5.docx]

**Supplemental Material 5. Annual trend of prevalence, incidence, and in-hospital mortality of cardiac amyloidosis**

| **Variables** | **2009** | **2010** | **2011** | **2012** | **2013** | **2014** | **2015** | **2016** | **2017** | **2018** | **2019** | **2020** |
| --- | --- | --- | --- | --- | --- | --- | --- | --- | --- | --- | --- | --- |
| **Number of subjects** | 75 | 91 | 112 | 164 | 191 | 239 | 283 | 339 | 398 | 441 | 499 | 560 |
| Prevalence^*^ (95%CI) | 0.20 (0.16–0.25) | 0.23 (0.19–0.29) | 0.28 (0.23–0.34) | 0.41 0.35–0.4) | 0.47 (0.41–0.55) | 0.59 (0.52–0.67) | 0.69 (0.61–0.77) | 0.81 (0.73–0.91) | 0.95 (0.86–1.05) | 1.04 (0.10–1.14) | 1.17 (1.07–1.28) | 1.30 (1.20–1.42) |
| **Number of incidences** | 33 | 29 | 29 | 56 | 45 | 65 | 59 | 82 | 86 | 86 | 92 | 96 |
| Incidence rate^*^ (95%CI) | 0.09 (0.06–0.12) | 0.07 (0.05–0.11) | 0.07 (0.05–0.11) | 0.14 (0.11–0.18) | 0.11 (0.08–0.15) | 0.16 (0.12–0.20) | 0.14 (0.11–0.19) | 0.20 (0.16–0.24) | 0.20 (0.16–0.25) | 0.20 (0.16–0.25) | 0.22 (0.17–0.26) | 0.22 (0.18–0.27) |
| **Number of In-hospital Mortality** | 13 | 8 | 4 | 18 | 17 | 15 | 26 | 27 | 43 | 34 | 35 | 34 |
| In-hospital mortality %^b,*^ (95%CI) | 17.3 (9.23–29.6) | 8.8 (3.80–17.3) | 3.6 (0.97–9.14) | 11.0 (6.50–17.4) | 8.9 (5.19–14.3) | 6.3 (3.51–10.4) | 9.2 (6.00–13.5) | 8.0 (5.25–11.6) | 10.8 (7.82–14.6) | 7.7 (5.34–10.8) | 7.0 (4.89–9.76) | 6.1 (4.21–8.48) |
| 1-year in-hospital mortality (%)^a^ | 12 (36.4) | 7 (24.1) | 4 (13.8) | 6 (10.7) | 10 (22.2) | 10 (15.4) | 15 (25.4) | 12 (14.6) | 15 (17.4) | 27 (31.4) | 22 (23.9) | 16 (16.7) |
| **AL-type cardiac amyloidosis** | |  |  |  |  |  |  |  |  |  |  |  |
| **Number of subjects** | 28 | 37 | 39 | 57 | 65 | 82 | 114 | 144 | 164 | 197 | 234 | 270 |
| Prevalence^*^ (95%CI) | 0.074 (0.049–0.160) | 0.095 (0.067–0.131) | 0.099 (0.070–0.135) | 0.143 (0.108–0.185) | 0.161 (0.125–0.206) | 0.201 (0.160–0.250) | 0.277 (0.228–0.332) | 0.346 (0.292–0.407) | 0.390 (0.333–0.455) | 0.465 (0.402–0.534) | 0.548 (0.480–0.623) | 0.627 (0.559–0.712) |
| **Number of incidences** | 13 | 13 | 6 | 20 | 15 | 26 | 35 | 44 | 31 | 47 | 49 | 54 |
| Incidence rate^*^ (95%CI) | 0.034 (0.018–0.058) | 0.033 (0.018–0.057) | 0.015 (0.006–0.003) | 0.050 (0.031–0.078) | 0.037 (0.208–0.614) | 0.064 (0.042–0.093) | 0.085 (0.059–0.118) | 0.106 (0.077–0.142) | 0.074 (0.050–1.047) | 0.111 (0.082–0.147) | 0.115 (0.085–0.152) | 0.125 (0.095–0.165) |
| **Number of in-hospital mortality** | 4 | 4 | 2 | 7 | 9 | 3 | 14 | 11 | 14 | 12 | 18 | 20 |
| Mortality, %^b,*^ (95%CI) | 14.3 (3.89–36.6) | 10.8 (2.95–27.7) | 5.1 (0.62–18.5) | 12.3 (4.94–25.3) | 13.9 (6.33–26.3) | 3.7 (0.75–10.7) | 12.3 (6.72–20.6) | 7.6 (3.81–13.7) | 8.5 (4.67–14.3) | 6.1 (3.15–10.6) | 7.7 (4.56–12.2) | 7.4 (4.53–11.4) |
| 1-year in-hospital mortality (%)^a^ | 3 (23.1) | 2 (15.4) | 3 (50.0) | 2 (10.0) | 4 (26.7) | 3 (11.5) | 9 (25.7) | 5 (11.4) | 6 (19.4) | 11 (23.4) | 9 (18.4) | 10 (18.5) |

Prevalence is expressed per 100,000 persons and incidence rate is presented as 100,000 person-years.

^a^ The number and proportion of deaths within the year among incident cases in each year. ^b^ The proportion of deaths among prevalent cases in each year. ^*^*P* for trend <0.0001.

CI, confidence interval.
