# Supplementary material for: Temporal trends in the prevalence, incidence, and mortality of cardiac amyloidosis in Korea over 12 years
Source: Epidemiol Health. 2024 Sep 15;46:e2024078. doi: 10.4178/epih.e2024078 (PMC11832237; doi:10.4178/epih.e2024078)
Supplement: Supplementary Material 6. — Trends in the prevalence and incidence of AL-type cardiac amyloidosis in the Korean population. [file epih-46-e2024078-Supplementary-6.docx]

**
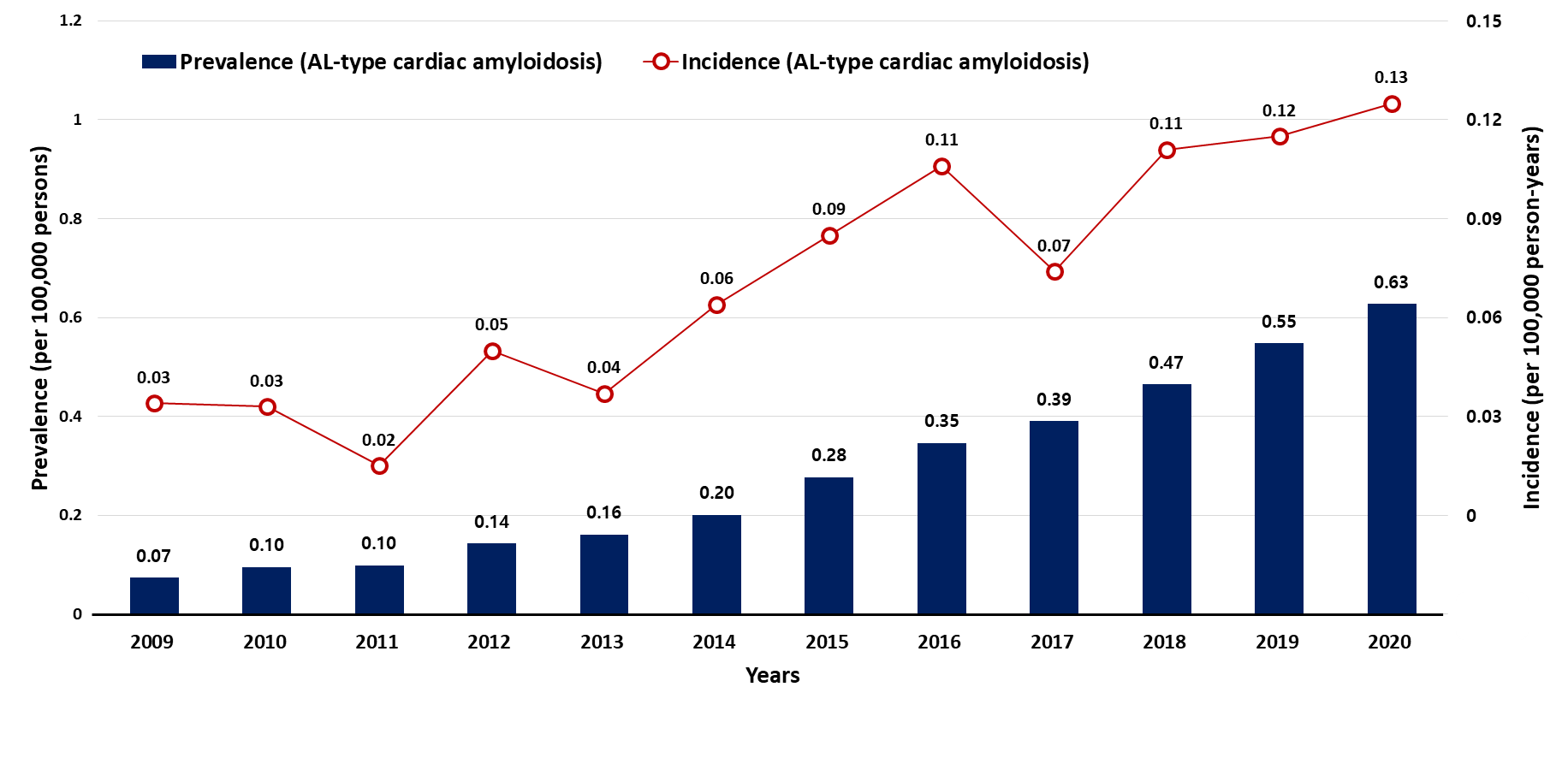
**

**Supplemental Material 6. Trends in the prevalence and incidence of AL-type cardiac amyloidosis in the Korean population.**

The bar graph displays the prevalence of cardiac amyloidosis (per 100,000 persons), and the line graph indicates the incidence of AL-type cardiac amyloidosis (per 100,000 person-years) in the Korean population between 2009 and 2020.
