# Supplementary material for: Temporal trends in the prevalence, incidence, and mortality of cardiac amyloidosis in Korea over 12 years
Source: Epidemiol Health. 2024 Sep 15;46:e2024078. doi: 10.4178/epih.e2024078 (PMC11832237; doi:10.4178/epih.e2024078)
Supplement: Supplementary Material 7. — Annual trend of prevalence, incidence, and in-hospital mortality of amyloidosis. [file epih-46-e2024078-Supplementary-7.docx]

**Supplemental Material 7. Annual trend of prevalence, incidence, and in-hospital mortality of amyloidosis.**

| **Variables** | **2009** | **2010** | **2011** | **2012** | **2013** | **2014** | **2015** | **2016** | **2017** | **2018** | **2019** | **2020** |
| --- | --- | --- | --- | --- | --- | --- | --- | --- | --- | --- | --- | --- |
| **Number of subjects** | 420 | 499 | 587 | 725 | 845 | 999 | 1,142 | 1,320 | 1,484 | 1,639 | 1,752 | 1,879 |
| Prevalence^*^  (95%CI) | 1.10 (1.00–1.22) | 1.28 (1.17–1.40) | 1.49 (1.37–1.62) | 1.82 (1.69–1.96) | 2.09 (1.96–2.24) | 2.45 (2.03–2.61) | 2.77 (2.61–2.94) | 3.17 (3.00–3.35) | 3.53 (3.35–3.71) | 3.87 (3.68–4.06) | 4.10 (3.91–4.30) | 4.36 (4.17–4.57) |
| **Number of incidences** | 161 | 112 | 129 | 162 | 161 | 214 | 192 | 241 | 232 | 235 | 196 | 204 |
| Incidence rate^*^  (95%CI) | 0.42 (0.36–0.49) | 0.29 (0.24–0.35) | 0.33 (0.27–0.39) | 0.41 (0.35–0.47) | 0.40 (0.34–0.47) | 0.53 (0.46–0.60) | 0.47 (0.40–0.54) | 0.58 (0.51–0.66) | 0.55 (0.48–0.63) | 0.55 (0.49–0.63) | 0.46 (0.40–0.53) | 0.47 (0.41–0.54) |
| **Number of in-hospital mortality** | 33 | 41 | 25 | 41 | 60 | 49 | 62 | 68 | 80 | 83 | 77 | 63 |
| In-hospital mortality (%)^b,*^ (95%CI) | 7.86 (5.41–11.0) | 8.22 (5.90–11.1) | 4.26 (2.76–6.29) | 5.66 (4.06–7.67) | 7.10 (5.42–9.14) | 4.90 (3.63–6.49) | 5.43 (4.16–6.96) | 5.15 (4.00–6.53) | 5.39 (4.28–6.71) | 5.06 (4.03–6.28) | 4.40 (3.47–5.49) | 3.35 (2.58–4.29) |
| 1-year in-hospital mortality (%)^a^ | 20 (12.4) | 26 (23.2) | 19 (14.7) | 29 (17.9) | 27 (16.8) | 38 (17.8) | 34 (17.7) | 36 (14.9) | 43 (18.5) | 41 (17.4) | 27 (13.8) | 33 (16.2) |
| **AL type amyloidosis** |  |  |  |  |  |  |  |  |  |  |  |  |
| **Number of subjects** | 155 | 175 | 207 | 260 | 308 | 358 | 443 | 527 | 587 | 676 | 738 | 806 |
| Prevalence^*^  (95%CI) | 0.407 (0.346–0.477) | 0.450 (0.385–0.521) | 0.526 (0.457–0.602) | 0.653 (0.576–0.737) | 0.764 (0.682–0.855) | 0.879 (0.790–0.975) | 1.075 (0.977–1.180) | 1.265 (1.160–1.378) | 1.396 (1.286–1.514) | 1.595 (1.477–1.720) | 1.727 (1.605–1.857) | 1.872 (1.745–2.005) |
| **Number of incidences** | 61 | 33 | 43 | 59 | 59 | 76 | 96 | 114 | 92 | 119 | 96 | 108 |
| Incidence rate^*^  (95%CI) | 0.160 (0.123–0.206) | 0.085 (0.058–0.119) | 0.109 (0.079–0.147) | 0.148 (0.113–0.191) | 0.146 (0.112–0.189) | 0.187 (0.147–0.233) | 0.233 (0.189–0.285) | 0.274 (0.226–0.329) | 0.219 (0.176–0.268) | 0.281 (0.233–0.336) | 0.225 (0.182–0.274) | 0.251 (0.206–0.303) |
| **Number of in-hospital mortality** | 13 | 11 | 6 | 11 | 26 | 11 | 30 | 32 | 30 | 34 | 40 | 33 |
| In-hospital mortality (%)^b,*^ (95%CI) | 8.39 (4.47–14.3) | 6.29 (3.14–11.3) | 2.90 (1.06–6.31) | 4.23 (2.11–7.57) | 8.44 (5.51–12.4) | 3.07 (1.53–5.50) | 6.77 (4.57–9.67) | 6.07 (4.15–8.57) | 5.11 (3.45–7.30 | 5.03 (3.48–7.03) | 5.42 (3.87–7.38) | 4.09 (2.82–5.75) |
| 1-year in-hospital mortality (%)^a^ | 8 (13.1) | 5 (15.2) | 5 (11.6) | 7 (11.9) | 9 (15.3) | 8 (10.5) | 14 (14.6) | 16 (14.0) | 13 (14.1) | 17 (14.3) | 12 (12.5) | 21 (19.4) |

Prevalence is expressed per 100,000 persons and incidence rate is presented as 100,000 person-years.

^a^ The number and proportion of deaths within the year among incident cases in each year. ^b^ The number and proportion of deaths among prevalent cases in each year. ^*^*P* for trend <0.0001.

CI, confidence interval.
