# Supplementary material for: Temporal trends in the prevalence, incidence, and mortality of cardiac amyloidosis in Korea over 12 years
Source: Epidemiol Health. 2024 Sep 15;46:e2024078. doi: 10.4178/epih.e2024078 (PMC11832237; doi:10.4178/epih.e2024078)
Supplement: Supplementary Material 8. — Kaplan–Meier curve for mortality in patients diagnosed with amyloidosis in Korea. [file epih-46-e2024078-Supplementary-8.docx]

**
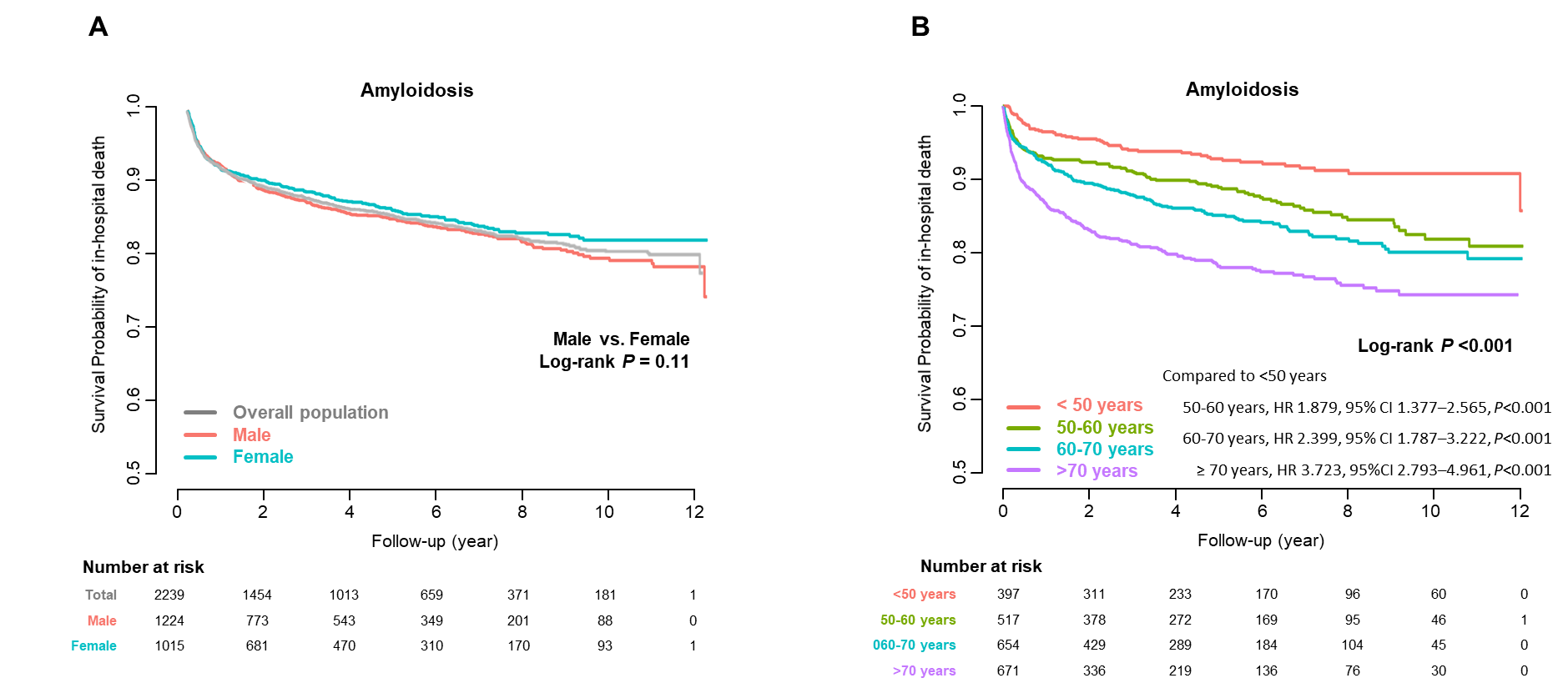
**

**Supplemental Material 8.** **Kaplan–Meier curve for mortality in patients diagnosed with amyloidosis in Korea.**

(A) The grey line represents the overall survival curve, whereas the pink and blue lines represent the survival curves for males and females, respectively. (B) Kaplan–Meier curves for overall mortality according to age group.
